# Associations Between Traumatic Brain Injury Characteristics and Memory Outcomes: Insights from the Health and Retirement Study

**DOI:** 10.3390/ijerph22020150

**Published:** 2025-01-23

**Authors:** Eric S. Cerino, Monica R. Lininger, Thomasina J. Seaton, Gillian Porter, Julie A. Baldwin

**Affiliations:** 1 Department of Psychological Sciences, Northern Arizona University, Flagstaff, AZ 86011, USA; tjs597@nau.edu; 2 Center for Health Equity Research, Northern Arizona University, Flagstaff, AZ 86011, USA; monica.lininger@nau.edu (M.R.L.); julie.baldwin@nau.edu (J.A.B.); 3 Department of Physical Therapy and Athletic Training, Northern Arizona University, Flagstaff, AZ 86011, USA; 4 Department of Occupational Therapy, Northern Arizona University, Phoenix, AZ 85004, USA; gillian.porter@nau.edu; 5 Department of Health Sciences, Northern Arizona University, Flagstaff, AZ 86011, USA

**Keywords:** traumatic brain injury, cognitive health, memory, preclinical dementia risk factors, preventative health

## Abstract

Traumatic brain injury (TBI) is an established risk factor for accelerated cognitive decline and increased dementia risk. The specific characteristics of TBI (e.g., type of head trauma, presence of a gap in memory, age of onset) that confer the greatest risk to cognitive health remain comparatively less clear. Using data from the 2014 Health and Retirement Study (HRS) experimental module, we examined associations between TBI characteristics and memory outcomes in a national adult lifespan sample. We tested whether the age of onset and presence of a memory gap in TBI resulting from a vehicle accident, from a fall or being hit, or from playing sports or playing on a playground were associated with self-rated memory and recall memory performance in a subsample of HRS respondents across the adult lifespan (*N* = 414, mean age = 66.28, SD = 9.70, 52% female). In cases where participants reported TBI from three different types of injury (vehicle accident, fall, and playing sports or playing on a playground), they shared whether they experienced a gap in their memory and their age when the head trauma occurred. Participants also reported on self-rated memory and performed a recall memory task. Hierarchical linear regression models were adjusted for age, sex, race, ethnicity, education, and self-rated health. Older age of onset for TBI from a fall was associated with worse self-rated memory (Est. = −0.11, SE = 0.04, *p* = 0.01) and recall performance (Est. = −0.33, SE = 0.15, *p* = 0.03). Encountering a memory gap from the TBI that resulted from a vehicle accident (Est. = −0.22, SE = 0.10, *p* = 0.03), a fall (Est. = −0.23, SE = 0.09, *p* = 0.01), and from playing sports or playing on a playground (Est. = −0.40, SE = −0.13, *p* < 0.01) were all significantly associated with worse self-rated memory. Links between encountering a memory gap and recall performance were comparatively scant. Results indicate the impact of TBI on memory varies as a function of type of trauma, age of onset, and presence of memory gap from the head trauma. Our study takes a preclinical, preventative approach to inform public health efforts that target the mitigation of specific types of head trauma at different developmental phases of the lifespan.

## 1. Introduction

### 1.1. Background of Traumatic Brain Injuries and Dementia

Traumatic brain injuries (TBIs) are becoming more common and are even referred to as a ‘silent epidemic’ [1]; however, the exact rates are challenging to estimate due to underreporting and non-disclosure by individuals following head trauma [2,3]. Recent estimates suggest that self-reported concussions during a lifetime have increased from 19.5% in 2016 to 24.7% in 2020 [4], with some researchers reporting prevalence as high as 28.9% [5]. Most hospitalizations in the United States (US) for TBI are caused by falls (49%) and motor vehicle accidents (25%) [6]. The vast majority of TBIs do not lead to hospitalization; they are treated through Emergency Departments (EDs), urgent care locations, and physician offices. Rates for those seen in the ED are typically due to falls (48%) or being struck by an external object, as seen in sports (17%) [7]. Long-term cognitive health risks from TBI (e.g., accelerated cognitive decline, increased dementia risk) compound this burden on public health and necessitate a preclinical, preventative approach to examining associations between TBI characteristics and memory outcomes in adulthood.

An estimated 6.5 million Americans are currently living with dementia, impeding health and well-being for the individuals diagnosed, caregivers, families, and communities across the US [8]. Identifying modifiable risk factors and protective factors for dementia is crucial to inform approaches for dementia prevention. Reducing modifiable risk factors and increasing modifiable protective factors of dementia can prevent or delay up to 45% of dementia cases [9]. Indeed, the 2024 report of the Lancet Commission conducted new meta-analyses that identified 14 dementia risk factors across developmental phases of the lifespan (early life, midlife, and later life). TBI was identified as a key risk factor that, if eliminated in midlife, could lead to a 3% reduction in dementia prevalence [9].

The association between dementia and TBI has been documented, but reports of the severity of this association are mixed [10,11,12]. Some researchers suggest that there is nearly a 70% increased risk of dementia following a TBI [13], increased dementia risk (hazard ratio of 1.51) following hospitalization due to a major TBI [14], and a 1.68 times greater risk of dementia following a TBI when accounting for sociodemographic characteristics and comorbidities [15]. Iacono and colleagues found that prior TBI can also serve as a significant risk factor for earlier age of onset for cognitive decline (i.e., an ‘age-lowering’ effect of TBI was observed independent of demographic factors, education, APOE genotype, and clinical conditions) [16]. However, other groups have found no association between dementia and TBI [11,12,17,18]. While increased evidence is seen assessing the connection between dementia and TBI, less is known about the preclinical factors, such as cognition or memory loss following head trauma later in life [19].

### 1.2. Present Study

While immediate memory loss can be a symptom following TBI [20,21], and cognitive tests are recommended after head trauma [20], the literature on the relationship between TBI and cognitive outcomes across the lifespan is limited. Currently, most of the literature is focused on older individuals (65 years of age), showing an increase in poor cognitive performance at three months following a mild traumatic brain injury (mTBI) when comparing a clinical cohort with a non-injured community-dwelling sample [19,22]. Recently, Lennon et al. [23] found that cognitive-related deficiencies did not worsen from baseline data collection throughout the 4-year study. Significant differences were noted at the start of the study, however, for those with prior TBI compared to those without, even with the traumatic head event occurring on average 30 years prior. In another study, no association was reported between self-reported lifetime history of TBI and a loss of consciousness with memory decline [24] or dementia with Lewy bodies [25]. In summary, the literature assessing the association between head trauma and memory loss in middle-aged adults is lacking.

Another consideration is the age at which TBI occurred in one’s life, which also presents conflicting results. Some scholars have found that the risk for dementia is higher when the traumatic head event occurs prior to 65 years of age [9,26]. However, others have found that older individuals with a mild TBI were at higher risk for dementia than those who were younger [10]. Also, Fann et al. [27] found that dementia risk was the lowest when there was a long timeframe between trauma and diagnosis, postulating that trauma earlier in life may not necessarily increase risk. The variability in how age is operationalized between research teams, however, limits effective comparisons within the literature. Further, research tends to focus on the assessment between the age of head trauma and diagnosis (e.g., dementia, Alzheimer’s), highlighting the need for investigation of preclinical factors like self-rated and performance-based memory outcomes.

Due to the lack of empirical evidence specific to TBI-related characteristics (e.g., type of head trauma, presence of a gap in memory, and age of onset), we examined the association of memory outcomes and these TBI descriptors in a cohort of adults from the 2014 Health and Retirement Study (HRS) experimental module. We hypothesized that older age of onset would be associated with worse memory outcomes. Further, we hypothesized that participants who reported having a gap in memory following their head trauma would report worse memory outcomes. We tested these associations in head traumas following a vehicle accident, fall, or sports-related injury.

## 2. Materials and Methods

### 2.1. Participants and Procedure

**Figure 1:**
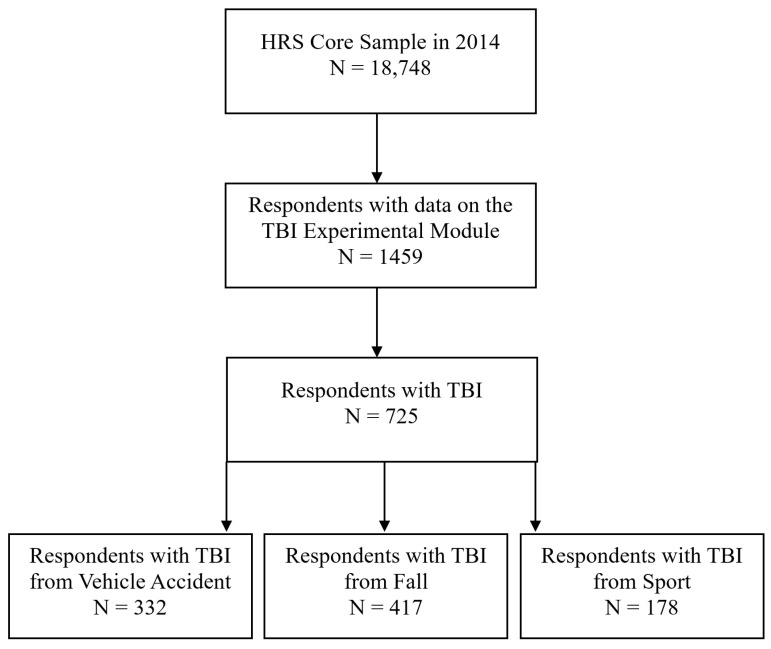
Sampling of respondents with data from the TBI experimental module from the 2014 core sample of the HRS. Respondents could have reported multiple TBI sources (i.e., Ns of 332, 417, and 178 are non-exclusive sums for respondents with TBI from a vehicle accident, fall, and sport, respectively). Specifically, 52 participants reported TBI from all three sources, 102 participants reported TBI from both a vehicle accident and fall, 10 participants reported TBI from both a vehicle accident and sports-related event, and 65 participants reported TBI from both a fall and sports-related event).

We conducted our secondary data analysis using a random de-identified subsample of the Health and Retirement Study (HRS) who completed an experimental module on TBI in 2014 (see https://hrs.isr.umich.edu/documentation/modules#2014 accessed on 25 February 2024) in addition to tests and surveys on cognitive health and well-being. The HRS is a longitudinal panel study of adults 50 years of age and older in the US, initiated in 1992. The 2–3 min experimental module on TBI was administered to approximately a 10% random sample of the core sample in 2014, resulting in a sample of 1459 respondents. Of these 1459 respondents, 725 reported a past head trauma and comprised our analytic sample (*N* = 725, mean age = 66.27 years, SD = 9.70, range = 50–99; 52% female; 11% Hispanic; 25% nonwhite; 34% with at least some college-level education or higher). See Figure 1 for a visual breakdown of the sampling of respondents. Seventeen participants (2%) reported a prior diagnosis of dementia, and two participants (<1%) reported a prior diagnosis of Alzheimer’s disease.

### 2.2. Measures

TBI Experimental Module. Participants were asked whether they had experienced six different types of injuries to their head or neck throughout their lives. Questions pertained to head trauma resulting from a vehicle accident (*n* = 332), from a fall or from being hit by something (*n* = 417), from playing sports or playing on a playground (*n* = 178), from being in a fight or hit by someone or shaken violently (*n* = 126), from being shot in the head (*n* = 5), and from being nearby when an explosion or a blast occurred (*n* = 153). We examined the TBI characteristics in injuries from a vehicle accident, from a fall or from being hit by something, and from playing sports or playing on a playground to maximize the sample size for statistical analysis and discuss public health recommendations within the context of the three most commonly reported head traumas in the HRS.

If a participant reported a specific head trauma, additional questions were asked, including their age when the injury occurred and the presence of a memory gap. The age of onset was operationalized as the age when the injury occurred (in years). On average, head traumas occurred in younger adulthood (vehicle: M = 31.67 years, SD = 18.38, range = 1–84; fall: M = 30.06 years, SD = 23.40, range = 1–89; sports-related: M = 15.94 years, SD = 9.76, range = 3–60). The age of onset was centered at the grand mean for each type of TBI to facilitate meaningful interpretation of parameter estimates using the sample average as the reference point. The presence of a memory gap was operationalized with a dichotomous response (0 = No, 1 = Yes) to the question “Were you dazed, or did you have a gap in your memory from this?” A memory gap was present in 43% of vehicle accidents, 50% of falls, and 49% of sports-related injuries.

Memory Outcomes. Self-rated memory was assessed with a single item asking participants to rate their memory at the time of survey completion (“How would you rate your memory at the present time?”) on a 5-point Likert-type scale (0 = poor, 1 = fair, 2 = good, 3 = very good, 4 = excellent; mean = 1.98, SD = 0.90, range = 0–4). Performance-based memory was assessed with a recall task where participants were instructed to list as many words as they remember from a list of 10 words immediately and after a delay (mean = 9.91 words, SD = 3.42, range = 0–18 words). Higher values indicated better cognitive health for both self-rated and recall memory.

Covariates. Participant age, sex, race, education, and self-rated health were included as covariates to adjust for sample heterogeneity. Chronological age was centered at the grand mean. Sex (0 = male, 1 = female), race (0 = white, 1 = nonwhite), ethnicity (0 = non-Hispanic, 1 = Hispanic), and education (0 = high school degree or less, 1 = some college-level education or higher) were coded as dichotomous variables. Self-rated health was assessed with a single item asking participants to rate their overall health (“Would you say your health is…”) on a 5-point Likert-type scale (0 = poor, 1 = fair, 2 = good, 3 = very good, 4 = excellent). Self-rated health was centered at the grand mean. Higher values indicated better self-rated health (mean = 2.07, SD = 1.07, range = 0–4).

### 2.3. Analytic Strategy

We conducted descriptive statistics and bivariate correlations to characterize the sample and describe associations across primary study variables before the primary and sensitivity analysis.

Primary Analysis. We then used hierarchical linear regression analyses to determine whether the TBI characteristics (i.e., age of onset, presence of memory gap) were associated with memory outcomes (i.e., self-rated memory, recall memory performance). First, we regressed the memory outcome on each TBI characteristic in unadjusted models. Next, we added the sociodemographic and health covariates previously discussed for the adjusted models to control for sample heterogeneity. Separate models estimated associations with self-rated memory and recall memory performance. These unadjusted and adjusted regression models were repeated three times for each type of head trauma (vehicle, fall, and sports-related), including model fit estimates of R square and Omega-square.

Sensitivity Analysis. The primary analysis included all participants who provided information on their TBI characteristics. This included approximately 3% of the analytic sample that reported a prior diagnosis of dementia or Alzheimer’s disease. We also conducted a sensitivity analysis that excluded these seventeen participants with a previous diagnosis of dementia (2%) and two participants with a prior diagnosis of Alzheimer’s disease (<1%) to remove any confound that neurodegenerative symptoms and disease may impose on the study findings. All analytic procedures were conducted in SAS (version 9.4).

## 3. Results

Table 1 provides descriptive statistics and bivariate correlations for primary study variables. Briefly, the cohort was nearly an equal split of males and females, primarily white (75%), with a third having some level of college education (34%) and in overall self-rated ‘good’ health. The following subsections describe hierarchical regression analysis results for self-rated and recall memory performance outcomes. Age of onset estimates are provided in SD units to interpret the association between age of onset and memory outcomes for a one SD difference in age of onset. The presence of memory gap estimates is interpreted in comparison to people who did not have a memory gap from their TBI.

### 3.1. Associations Between TBI Age of Onset and Memory Outcomes

#### 3.1.1. TBI Age of Onset and Self-Rated Memory

**Figure 2:**
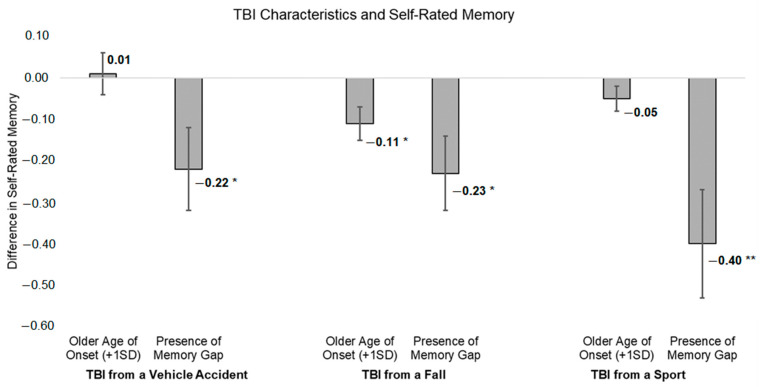
Associations between TBI characteristics and self-rated memory, including effect size estimates for differences. * *p* < 0.05, ** *p* < 0.01.

Older age of onset for TBI from a fall was associated with significantly worse self-rated memory (Est. = −0.11, SE = 0.04, *p* = 0.01) in the initial unadjusted model. When adjusting for sociodemographic and health variables (i.e., age, sex, race, education, self-rated health), this association attenuated to non-significance (Est. = −0.06, SE = 0.04, *p* = 0.12). Age of onset for TBI from a vehicle accident was not associated with self-rated memory (unadjusted model: Est. = 0.01, SE = 0.05, *p* = 0.88; adjusted model: Est. = −0.01, SE = 0.05, *p* = 0.86). Similarly, age of onset for TBI from a sports-related injury was also not associated with self-rated memory (unadjusted model: Est. = −0.05, SE = 0.04, *p* = 0.20; adjusted model: Est. = −0.05, SE = 0.03, *p* = 0.13). Full-model reporting is included in Table 2 with visualization of estimates from initial models displayed in Figure 2.

#### 3.1.2. TBI Age of Onset and Recall Memory Performance

The pattern of results was similar for recall memory performance. Older age of onset for TBI from a fall was associated with significantly worse recall memory performance (Est. = −0.33, SE = 0.15, *p* = 0.03) in the initial unadjusted model. This association attenuated to non-significance when adjusting for sociodemographic and health variables (Est. = −0.09, SE = 0.14, *p* = 0.53).

**Figure 3:**
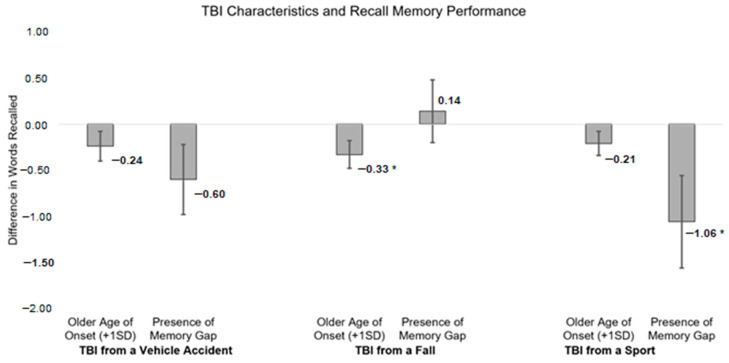
Associations between TBI characteristics and recall memory performance, including effect size estimates for differences. * *p* < 0.05.

Age of onset for TBI from a vehicle accident (unadjusted model: Est. = −0.24, SE = 0.16, *p* = 0.13; adjusted model: Est. = −0.04, SE = 0.15, *p* = 0.78) and from a sports-related injury (unadjusted model: Est. = −0.21, SE = 0.13, *p* = 0.11; adjusted model: Est. = −0.15, SE = 0.12, *p* = 0.21) was not associated with recall memory performance. Full-model reporting is included in Table 3 with visualization of estimates from initial models displayed in Figure 3.

### 3.2. Associations Between Presence of Memory Gap from TBI and Memory Outcomes

#### 3.2.1. Presence of Memory Gap and Self-Rated Memory

Encountering a TBI memory gap resulting from a sports-related injury was associated with significantly worse self-rated memory (Est. = −0.40, SE = 0.13, *p* < 0.01) in the initial unadjusted model. This association held after adjusting for sociodemographic and health variables (Est. = −0.41, SE = 0.13, *p* < 0.01).

Encountering a memory gap from the TBI that resulted from a vehicle accident (Est. = −0.22, SE = 0.10, *p* = 0.03) and from a fall (Est. = −0.23, SE = 0.09, *p* = 0.01) was associated with significantly worse self-rated memory in initial unadjusted models. However, the association between the presence of a memory gap and worse self-rated memory attenuated to non-significance when adjusting for sociodemographic and health variables for both the vehicle (Est. = −0.14, SE = 0.10, *p* = 0.15) and fall (Est. = −0.13, SE = 0.09, *p* = 0.14) models. Full-model reporting is included in Table 4 with visualization of estimates from initial models displayed in Figure 2.

#### 3.2.2. Presence of Memory Gap and Recall Memory Performance

Encountering a TBI memory gap resulting from a sports-related injury was associated with significantly worse recall memory performance (Est. = −1.06, SE = 0.50, *p* = 0.03) in the initial unadjusted model. This association attenuated to non-significance when adjusting for sociodemographic and health variables (Est. = −0.67, SE = 0.47, *p* = 0.16).

The presence of a memory gap from the TBI that resulted from a vehicle accident (unadjusted model: Est. = −0.60, SE = 0.38, *p* = 0.11; adjusted model: Est. = −0.44, SE = 0.35, *p* = 0.21) and from a fall (unadjusted model: Est. = 0.14, SE = 0.34, *p* = 0.69; adjusted model: Est. = 0.45, SE = 0.31, *p* = 0.15) was not associated with recall memory performance. Full-model reporting is included in Table 5 with visualization of estimates from initial models displayed in Figure 3.

### 3.3. Sensitivity Analysis

We have included Supplemental Materials that provides full-model results for analysis among people without dementia (Tables S1–S4) and analysis among people without Alzheimer’s disease (Tables S5–S8). Results of this sensitivity analysis revealed that findings from primary analyses held when reducing the analytic sample to people without dementia or Alzheimer’s disease.

## 4. Discussion

In this study, we examined the association between two memory outcomes (e.g., self-rated memory and recall memory performance) with the age of onset for TBI and the presence of a memory gap (i.e., being dazed or having a gap in your memory from the head trauma) stratified by TBI-related characteristics (e.g., motor vehicle accident, fall, sport) in a cohort of community-dwelling adults. Our primary results suggest that self-rated memory was significantly worse when a memory gap occurred following a self-reported TBI caused by a sports-related event. This association was robust to the influence of sociodemographic and health factors. Older age of onset for TBI following a fall was related to worse self-rated memory and recall memory performance, but these associations attenuated to non-significance when adjusting for sociodemographic and health factors. Preclinical measures of self-rated memory and recall memory performance were more negatively impacted by a memory gap following the TBI than the age when the event occurred.

A deficit in memory following a TBI is a known symptom [20,21,28,29] but typically subsides within days to a few weeks [30]. In this cohort, the presence of a memory gap was associated with a worse self-rated memory score regardless of the type of TBI characteristic (vehicle accident, fall, or sports-related); however, this association was impacted by sociodemographic variables (e.g., age, sex, race, ethnicity, education) and self-rated health for TBI suffered from vehicle accidents and falls. In prior work, Whiteneck et al. [31] found that self-reported memory was poor for those with a prior TBI; however, in that study, the mechanism of TBI and a measure of health were not included in the analytic models. Sociodemographic variables have been shown to significantly impact cognition after sustaining a TBI. More specifically, females [32], Hispanic adults [33], and African American adults [34] have worse cognitive outcomes when compared to males, non-Hispanic adults, and white adults, respectively, following head trauma. These factors may explain the non-significant findings in the adjusted models of the present work.

In sports-related head traumas, the TBI and associated memory gap impacted self-rated memory regardless of possible social determinants of health, which could be due to the fairly homogeneous cohort. Another possibility is that repetitive subconcussive hits [35] could take place in sports, compared to the other more acute mechanisms of vehicle accidents and falls, which might be more impactful on self-rated memory than the included demographic variables. Additionally, chronic traumatic encephalopathy has been linked with repetitive head trauma [36], and an early-stage symptom is memory loss [37]. The relationship between a documented memory gap and impacted cognitive outcome in sports, regardless of sociodemographic and health factors, did not hold true for recall memory performance. This could be due to the more objective nature of the measure, which aligns with previous research in the HRS that found associations between history of TBI and worse self-rated memory but not objective cognitive performance [38]. Most of the literature focuses on memory outcomes immediately following the head trauma and then again at 3 months [19,22] or a year; the literature is lacking across the lifespan, thus indicating the novelty of the present work. The current findings extend past work to include multiple characteristics of TBI and preclinical cognitive health outcomes in a sample of community-dwelling adults across the adult lifespan.

Significant falls that led to a TBI later in life were related to worse self-rated memory and recall memory performance. That said, the association in the current study was no longer present when incorporating sociodemographic and health factors. This suggests the associations between the age of onset for TBI from a fall and memory outcomes may be partly due to structural differences in sociodemographic and health factors in the present sample. Future research is needed to more formally test the mechanisms underlying these associations and evaluate their role in shaping future dementia risk as people grow older.

Meta-analytic summaries of dementia research suggest TBI is a stronger risk factor for dementia among people of younger ages [9,26]. However, it is essential to note that past research on the role age plays in links between TBI and cognition is mixed and varies widely in how age is operationalized (e.g., age of onset, age at study baseline before or after TBI, age at time of outcome assessment) [13]. Further, most prior research focuses on the role of age in TBI when assessing dementia risk, leaving a critical gap in understanding the role age of onset for TBI may play in preclinical memory outcomes. The present study’s findings are consistent with prior research that similarly operationalized age as the person’s age when they endured the TBI [10,27]. For example, Gardner and colleagues [10] found that mild TBI (mTBI) was a stronger risk factor for dementia for people with older age of TBI onset. Further, Fann and colleagues [27] found dementia risk was lowest among those with more time since their TBI, making less time since TBI (and comparatively older age of onset) a significant risk factor for dementia. The present study extends this line of work to document links between older age of onset and self-rated memory and recall memory performance in a community-dwelling, preclinical sample of adults 50 years of age and older.

While this is a large population-based study of community-dwelling individuals, there are still limitations that should be noted. As with self-reported measures, recall bias is possible, especially when asking questions across a lifespan. Future research with closer assessment to the TBI event and subsequent longitudinal follow-up is needed to evaluate the predictive utility of TBI characteristics for assessing changes in self-rated memory and recall memory performance over time. Additionally, the analytic sample’s lack of diversity in racial and ethnic composition, as well as the lack of individuals in the lowest socioeconomic stratum, is a limitation when considering the generalizability of the present findings. With the population increasingly becoming diverse in socioeconomic, racial, and ethnic composition, it is crucial for future work to evaluate how associations between TBI characteristics and memory outcomes may vary within and across critical sociodemographic dimensions. Future research should also evaluate the extent to which other TBI characteristics (e.g., severity, number of TBIs) and individual differences in ADRD biomarkers such as β amyloid deposition, pathologic tau, and neurodegeneration [AT[N]] [39] may impact self-rated memory and performance-based memory indices.

Results from the present study build a clearer understanding of the role of TBI characteristics for preclinical memory outcomes and may inform public health efforts to promote dementia prevention across the adult lifespan. Specifically, the significant link between the older age of onset for TBI from a fall and memory outcomes specifies for whom (i.e., comparatively older adults) targeted cognitive health supports and monitoring would be most helpful following head trauma from a fall. Further, understanding the impact a memory gap following TBI from a sports-related injury can have on preclinical memory outcomes can inform better screening and documentation practices around TBI in sports to identify the characteristics of the TBI (i.e., memory gap) that are most robustly linked to memory outcomes. These improvements in cognitive monitoring can parallel other public health campaigns advocating for safe practices in sports that protect against head injury and work toward mitigating dementia risk (e.g., updates to head protection equipment, preventing play immediately after TBI, limiting high-impact collision [9]). Longitudinal follow-up is a critical next step for evaluating the roles age of onset and presence of memory gap have for possible transitions to dementia and the mechanisms underlying these associations.

## 5. Conclusions

The impact of traumatic brain injury on memory varies as a function of type of trauma, age of onset, and presence of memory gap from the head trauma. Our study takes a preclinical, preventative approach to inform public health efforts that target the mitigation of specific types of head trauma at different developmental phases of the lifespan.

## Figures and Tables

**Table 1:** Descriptive statistics and bivariate correlations for study variables.

Variable	M (SD)/%	1	2	3	4	5	6	7	8	9	10	11	12	13	14	15	16
1. Age	66.28 (9.70)	-															
2. Female (%)	0.52	−0.07 †	−														
3. Hispanic (%)	0.11	−0.19 ***	0.05	−													
4. Nonwhite (%)	0.25	−0.21 ***	0.001	0.15 ***	−												
5. Some College Education (%)	0.34	−0.04	0.01	−0.10 **	−0.15 ***	−											
6. Health	2.07 (1.07)	0.01	0.01	−0.08 *	−0.14 ***	0.17 ***	−										
7. Alzheimer’s Disease (%)	0.003	0.09 *	0.05	0.07 †	0.03	−0.04	−0.05	−									
8. Dementia (%)	0.02	0.10 **	0.04	−0.03	0.01	−0.02	−0.16 ***	0.34 *	−								
9. Age: Vehicle	31.67 (18.38)	0.32 ***	0.02	0.05	−0.05	0.03	−0.05	0.12 *	0.08	−							
10. Age: Fall	30.06 (23.40)	0.30 ***	0.14 **	−0.07	−0.10 *	−0.06	−0.13 *	0.13 **	0.10 *	0.44 ***	−						
11. Age: Sports	15.94 (9.76)	0.01	−0.06	0.19*	0.11	0.02	0.02	−	−0.01	0.21	0.40 ***	−					
12. MG: Vehicle (%)	0.43	−0.15 **	−0.17 *	0.01	0.05	−0.07	−0.08	−0.05	0.07	−0.02	−0.05	−0.07	−				
13. MG: Fall (%)	0.50	−0.10 *	−0.13 *	−0.02	0.03	−0.08 †	−0.16 **	−0.001	0.06	0.04	−0.04	0.17 †	0.50 ***	−			
14. MG: Sports (%)	0.49	−0.01	−0.17 *	0.06	0.10	−0.18 *	−0.06	0.08	0.08	0.08	0.21 *	0.05	0.62 ***	0.46 ***	−		
15. Self-Rated Memory	1.98 (0.90)	−0.04	0.01	0.003	−0.05	0.18 ***	0.32 ***	0.001	−0.12 **	0.07	−0.11 *	−0.09	−0.12 *	−0.13 *	−0.22 **	−	
16. Recall Memory	9.91 (3.42)	−0.25 ***	0.21 ***	−0.04	−0.09 *	0.29 ***	0.24 ***	−0.10 **	−0.12 **	−0.08	−0.10 *	−0.03	−0.09	0.02	−0.16 *	0.23 ***	-

Note. † *p* < 0.10, * *p* < 0.05, ** *p* < 0.01, *** *p* < 0.001. Abbreviations: M = mean, SD = standard deviation, MG = memory gap, health = self-reported health.

**Table 2:** Hierarchical regression analyses for TBI age of onset predicting self-rated memory.

Variable	TBI from a Vehicle Accident				TBI from a Fall				TBI from a Sport			
	Model 1: Unadjusted		Model 2: Sociodemographic and Health Adjustment		Model 1: Unadjusted		Model 2: Sociodemographic and Health Adjustment		Model 1: Unadjusted		Model 2: Sociodemographic and Health Adjustment	
	B (SE)	95% CI	B (SE)	95% CI	B (SE)	95% CI	B (SE)	95% CI	B (SE)	95% CI	B (SE)	95% CI
Intercept	2.02 (0.05) ***	[1.92, 2.12]	1.87 (0.09) ***	[1.70, 2.04]	1.96 (0.04) ***	[1.87, 2.05]	1.88 (0.08) ***	[1.73, 2.03]	1.98 (0.07) ***	[1.85, 2.12]	1.92 (0.11) ***	[1.70, 2.14]
Age (+1SD)			0.06 (0.05)	[−0.05, 0.16]			0.002 (0.04)	[−0.08, 0.09]			0.02 (0.08)	[−0.13, 0.17]
Female			0.02 (0.10)	[−0.17, 0.21]			−0.03 (0.09)	[−0.20, 0.14]			−0.04 (0.14)	[−0.32, 0.24]
Hispanic			−0.03 (0.15)	[−0.32, 0.27]			0.27 (0.13) *	[0.01, 0.53]			0.64 (0.30) *	[0.05, 1.22]
Nonwhite			0.13 (0.11)	[−0.09, 0.34]			0.06 (0.11)	[−0.15, 0.27]			0.04 (0.16)	[−0.27, 0.35]
Some College Education			0.34 (0.10) **	[0.14, 0.54]			0.17 (0.09) †	[−0.01, 0.35]			0.14 (0.14)	[−0.13, 0.42]
Health			0.26 (0.04) ***	[0.17, 0.34]			0.27 (0.04) ***	[0.19, 0.35]			0.31 (0.06) ***	[0.19, 0.43]
Age of Onset (+1SD)	0.01 (0.04)	[−0.07, 0.09]	−0.01 (0.04)	[−0.09, 0.07]	−0.11 (0.04) **	[−0.19, −0.03]	−0.06 (0.04)	[−0.14, 0.02]	−0.05 (0.04)	[−0.12, 0.02]	−0.05 (0.03)	[−0.12, 0.01]
Model Statistics												
F (DF)	0.02 (1, 325)		7.77 (7, 316) ***		6.96 (1, 410) **		9.25 (7, 404) ***		1.68 (1, 175)		5.41 (7, 167) ***	
R Square	0.0001		0.15		0.02		0.14		0.01		0.18	
Omega-Square	0.00	[0.00, 0.01]	0.13	[0.07, 0.20]	0.01	[0.00, 0.05]	0.12	[0.07, 0.19]	0.00	[0.00, 0.06]	0.15	[0.06, 0.26]

Note. † *p* < 0.10, * *p* < 0.05, ** *p* < 0.01, *** *p* < 0.001.

**Table 3:** Hierarchical regression analyses for TBI age of onset predicting recall memory performance.

Variable	TBI from a Vehicle Accident				TBI from a Fall				TBI from a Sport			
	Model 1: Unadjusted		Model 2: Sociodemographic and Health Adjustment		Model 1: Unadjusted		Model 2: Sociodemographic and Health Adjustment		Model 1: Unadjusted		Model 2: Sociodemographic and Health Adjustment	
	B (SE)	95% CI	B (SE)	95% CI	B (SE)	95% CI	B (SE)	95% CI	B (SE)	95% CI	B (SE)	95% CI
Intercept	10.04 (0.19) ***	[9.66, 10.41]	8.90 (0.32) ***	[8.27, 9.54]	9.99 (0.17) ***	[9.66, 10.32]	8.81 (0.27) ***	[8.28, 9.34]	10.01 (0.25) ***	[9.51, 10.50]	9.25 (0.40) ***	[8.47, 10.04]
Age (+1SD)			−0.78 (0.20) ***	[−1.17, −0.40]			−0.89 (0.16) ***	[−1.20, −0.58]			−0.55 (0.28) *	[−1.09, −0.002]
Female			1.07 (0.35) **	[0.38, 1.77]			1.14 (0.31) ***	[0.54, 1.75]			0.57 (0.50)	[−0.42, 1.56]
Hispanic			−0.59 (0.55)	[−1.68, 0.50]			−0.54 (0.48)	[−1.48, 0.40]			−0.74 (1.06)	[−2.83, 1.34]
Nonwhite			−1.03 (0.41) *	[−1.84, −0.23]			−0.58 (0.38)	[−1.32, 0.17]			−0.30 (0.55)	[−1.39, 0.79]
Some College Education			1.75 (0.38) ***	[1.001, 2.50]			1.74 (0.33) ***	[1.10, 2.38]			1.38 (0.49) **	[0.41, 2.35]
Health			0.66 (0.16) ***	[0.35, 0.98]			0.57 (0.15) ***	[0.28, 0.86]			0.98 (0.22) ***	[0.54, 1.41]
Age of Onset (+1SD)	−0.24 (0.16)	[−0.54, 0.07]	−0.04 (0.15)	[−0.34, 0.25]	−0.33 (0.15) *	[−0.63, −0.03]	−0.09 (0.14)	[−0.38, 0.19]	−0.21 (0.13)	[−0.47, 0.05]	−0.15 (0.12)	[−0.39, 0.09]
Model Statistics												
F (DF)	2.31 (1, 324)		12.04 (7, 315) ***		4.70 (1, 412) *		16.79 (7, 406) ***		2.53 (1, 175)		6.92 (7, 167) ***	
R Square	0.01		0.21		0.01		0.22		0.01		0.22	
Omega-Square	0.00	[0.00, 0.04]	0.19	[0.12, 0.27]	0.01	[0.00, 0.04]	0.21	[0.15, 0.28]	0.00	[0.00, 0.07]	0.19	[0.10, 0.30]

Note. * *p* < 0.05, ** *p* < 0.01, *** *p* < 0.001.

**Table 4:** Hierarchical regression analyses for presence of memory gap from TBI predicting self-rated memory.

Variable	TBI from a Vehicle Accident				TBI from a Fall				TBI from a Sport			
	Model 1: Unadjusted		Model 2: Sociodemographic and Health Adjustment		Model 1: Unadjusted		Model 2: Sociodemographic and Health Adjustment		Model 1: Unadjusted		Model 2: Sociodemographic and Health Adjustment	
	B (SE)	95% CI	B (SE)	95% CI	B (SE)	95% CI	B (SE)	95% CI	B (SE)	95% CI	B (SE)	95% CI
Intercept	2.12 (0.07) ***	[1.99, 2.25]	2.00 (0.10) ***	[1.80, 2.20]	2.07 (0.06) ***	[1.95, 2.20]	1.96 (0.09) ***	[1.78, 2.14]	2.20 (0.09) ***	[2.02, 2.38]	2.17 (0.13) ***	[1.91, 2.44]
Age (+1SD)			0.04 (0.05)	[−0.06, 0.14]			−0.02 (0.04)	[−0.11, 0.06]			−0.01 (0.08)	[−0.15, 0.14]
Female			−0.05 (0.10)	[−0.24, 0.14]			−0.07 (0.09)	[−0.24, 0.10]			−0.08 (0.14)	[−0.36, 0.19]
Hispanic			−0.07 (0.14)	[−0.35, 0.22]			0.27 (0.14) *	[0.002, 0.54]			0.63 (0.29) *	[0.06, 1.19]
Nonwhite			0.09 (0.11)	[−0.12, 0.31]			0.07 (0.11)	[−0.15, 0.28]			0.03 (0.15)	[−0.27, 0.33]
Some College Education			0.31 (0.10) **	[0.11, 0.51]			0.18 (0.09) †	[−0.003, 0.36]			0.07 (0.14)	[−0.20, 0.34]
Health			0.24 (0.04) ***	[0.16, 0.33]			0.27 (0.04) ***	[0.19, 0.35]			0.28 (0.06) ***	[0.16, 0.40]
Memory Gap	−0.22 (0.10) *	[−0.42, −0.03]	−0. 14 (0.10)	[−0.33, 0.05]	−0.23 (0.09) *	[−0.41, −0.05]	−0.13 (0.09)	[−0.30, 0.04]	−0.40 (0.13) **	[−0.66, 0.14]	−0.41 (0.13) **	[−0.67, −0.16]
Model Statistics												
F (DF)	4.93 (1, 320) *		7.51 (7, 311) ***		6.54 (1, 404) *		8.99 (7, 398) ***		9.17 (1, 173)		5.88 (7, 165) ***	
R Square	0.02		0.14		0.02		0.14		0.05		0.20	
Omega-Square	0.01	[0.00, 0.05]	0.13	[0.06, 0.20]	0.01	[0.00, 0.05]	0.12	[0.07, 0.19]	0.04	[0.01, 0.13]	0.16	[0.07, 0.27]

Note. † *p* < 0.10, * *p* < 0.05, ** *p* < 0.01, *** *p* < 0.001.

**Table 5:** Hierarchical regression analyses for presence of memory gap from TBI predicting recall memory performance.

Variable	TBI from a Vehicle Accident				TBI from a Fall				TBI from a Sport			
	Model 1: Unadjusted		Model 2: Sociodemographic and Health Adjustment		Model 1: Unadjusted		Model 2: Sociodemographic and Health Adjustment		Model 1: Unadjusted		Model 2: Sociodemographic and Health Adjustment	
	B (SE)	95% CI	B (SE)	95% CI	B (SE)	95% CI	B (SE)	95% CI	B (SE)	95% CI	B (SE)	95% CI
Intercept	10.37 (0.25) ***	[9.88, 10.86]	9.37 (0.37) ***	[8.64, 10.09]	9.93 (0.24) ***	[9.45, 10.40]	8.55 (0.33) ***	[7.91, 9.20]	10.53 (0.35) ***	[9.85, 11.22]	9.58 (0.48) ***	[8.63, 10.54]
Age (+1SD)			−0.90 (0.18) ***	[−1.26, −0.54]			−0.89 (0.15) ***	[−1.20, −0.59]			−0.55 (0.28) *	[−1.09, −0.01]
Female			0.84 (0.35) **	[0.15, 1.54]			1.13 (0.31) ***	[0.53, 1.74]			0.42 (0.50)	[−0.57, 1.42]
Hispanic			−0.51 (0.52)	[−1.53, 0.52]			−0.59 (0.48)	[−1.54, 0.36]			−0.80 (1.03)	[−2.84, 1.25]
Nonwhite			−1.28 (0.40) **	[−2.08, −0.49]			−0.55 (0.38)	[−1.30, 0.20]			−0.13 (0.55)	[−1.22, 0.95]
Some College Education			1.59 (0.37) ***	[0.86, 2.32]			1.87 (0.33) ***	[1.22, 2.52]			1.33 (0.49) **	[0.36, 2.30]
Health			0.61 (0.16) ***	[0.30, 0.92]			0.57 (0.15) ***	[0.28, 0.87]			1.02 (0.22) ***	[0.58, 1.46]
Memory Gap	−0.60 (0.38)	[−1.34, 0.15]	−0.44 (0.35)	[−1.14, 0.25]	0.14 (0.34)	[−0.53, 0.81]	0.45 (0.31)	[−0.16, 1.07]	−1.06 (0.50) *	[−2.04, −0.08]	−0.67 (0.47)	[−1.59, 0.26]
Model Statistics												
F (DF)	2.50 (1, 318)		12.27 (7, 309) ***		0.16 (1, 406) *		16.77 (7, 400) ***		4.58 (1, 173) *		7.42 (7, 165) ***	
R Square	0.01		0.22		0.00		0.23		0.03		0.24	
Omega-Square	0.00	[0.00, 0.04]	0.20	[0.13, 0.28]	0.00	[0.00, 0.01]	0.21	[0.15, 0.28]	0.02	[0.00, 0.09]	0.21	[0.11, 0.32]

Note. * *p* < 0.05, ** *p* < 0.01, *** *p* < 0.001.

## Data Availability

Data are publicly available at the following website: (https://hrs.isr.umich.edu/data-products, accessed on 25 February 2024). Study materials and the study analysis code may be made available for appropriate use upon emailed request to the corresponding authors.

## Supplementary Materials

The following supporting information can be downloaded at https://www.mdpi.com/article/10.3390/ijerph22020150/s1, Table S1: Hierarchical regression analyses for TBI age of onset predicting self-rated memory in people without dementia; Table S2: Hierarchical regression analyses for TBI age of onset predicting recall memory performance in people without dementia; Table S3: Hierarchical regression analyses for presence of memory gap from TBI predicting self-rated memory in people without dementia; Table S4: Hierarchical regression analyses for presence of memory gap from TBI predicting recall memory performance in people without dementia; Table S5: Hierarchical regression analyses for TBI age of onset predicting self-rated memory in people without Alzheimer’s disease; Table S6: Hierarchical regression analyses for TBI age of onset predicting recall memory performance in people without Alzheimer’s disease; Table S7: Hierarchical regression analyses for presence of memory gap from TBI predicting self-rated memory in people without Alzheimer’s disease; Table S8: Hierarchical regression analyses for presence of memory gap from TBI predicting recall memory performance in people without Alzheimer’s disease.
