# Supplementary material for: Associations Between Traumatic Brain Injury Characteristics and Memory Outcomes: Insights from the Health and Retirement Study
Source: Int J Environ Res Public Health. 2025 Jan 23;22(2):150. doi: 10.3390/ijerph22020150 (PMC11855548; doi:10.3390/ijerph22020150)
Supplement: Supplementary file 1 [file ijerph-22-00150-s001.zip › ijerph-3397277-supplementary.pdf]

Supplementary Material

Table S1. Hierarchical regression analyses for TBI age of onset predicting self−rated memory in people without dementia.

**Variable**	**TBI from a Vehicle Accident**				**TBI from a Fall**				**TBI from a Sport**			
	**Model 1: Unadjusted**		**Model 2: Sociodemographic and Health Adjustment**		**Model 1: Unadjusted**		**Model 2: Sociodemographic and Health Adjustment**		**Model 1: Unadjusted**		**Model 2: Sociodemographic and Health Adjustment**	
	**B (SE)**	**95% CI**	**B (SE)**	**95% CI**	**B (SE)**	**95% CI**	**B (SE)**	**95% CI**	**B (SE)**	**95% CI**	**B (SE)**	**95% CI**
Intercept	2.03 (0.05) ***	[1.94, 2.13]	1.88 (0.09) ***	[1.71, 2.05]	2.00 (0.04) ***	[1.91, 2.09]	1.94 (0.08) ***	[1.79, 2.09]	2.00 (0.07) ***	[1.87, 2.14]	1.90 (0.11) ***	[1.68, 2.13]
Age (+1SD)			0.04 (0.05)	[−0.06, 0.15]			−0.004 (0.04)	[−0.09, 0.08]			−0.04 (0.08)	[−0.20, 0.13]
Female			0.02 (0.10)	[−0.17, 0.21]			−0.06 (0.09)	[−0.23, 0.11]			−0.02 (0.14)	[−0.30, 0.27]
Hispanic			−0.04 (0.15)	[−0.33, 0.25]			0.24 (0.13) †	[−0.02, 0.50]			0.60 (0.30) *	[0.01, 1.19]
Nonwhite			0.13 (0.11)	[−0.10, 0.35]			0.02 (0.11)	[−0.19, 0.24]			0.01 (0.16)	[−0.30, 0.32]
Some College			0.33 (0.10) **	[0.13, 0.53]			0.16 (0.09) †	[−0.02, 0.34]			0.15 (0.14)	[−0.13, 0.42]
Health			0.25 (0.04) ***	[0.17, 0.34]			0.26 (0.04) ***	[0.17, 0.34]			0.29 (0.06) ***	[0.17, 0.42]
Age of Onset (+1SD)	−0.01 (0.04)	[−0.09, 0.07]	−0.02 (0.04)	[−0.10, 0.06]	−0.09 (0.04) *	[−0.17, −0.01]	−0.05 (0.04)	[−0.13, 0.03]	−0.01 (0.04)	[−0.09, 0.07]	−0.04 (0.04)	[−0.12, 0.03]
Model Statistics												
F (DF)	0.03 (1, 315)		7.15 (7, 306) ***		4.61 (1, 395) *		7.77 (7, 389) ***		0.10 (1, 169)		4.36 (7, 161) ***	
R Square	0.0001		0.14		0.01		0.12		0.001		0.16	
Omega−Square	0.00	[0.00, 0.01]	0.12	[0.06, 0.20]	0.01	[0.00, 0.04]	0.11	[0.06, 0.17]	0.01	[0.00, 0.03]	0.12	[0.04, 0.23]

Note. † $p < 0.10$, * $p < 0.05$, ** $p < 0.01$, *** $p < 0.001$.

Table S2. Hierarchical regression analyses for TBI age of onset predicting recall memory performance in people without dementia.

Variable	**TBI from a Vehicle Accident**				**TBI from a Fall**				**TBI from a Sport**			
	**Model 1: Unadjusted**		**Model 2: Sociodemographic and Health Adjustment**		**Model 1: Unadjusted**		**Model 2: Sociodemographic and Health Adjustment**		**Model 1: Unadjusted**		**Model 2: Sociodemographic and Health Adjustment**	
	**B(SE)**	**95% CI**	**B (SE)**	**95% CI**	**B (SE)**	**95% CI**	**B (SE)**	**95% CI**	**B (SE)**	**95% CI**	**B (SE)**	**95% CI**
Intercept	10.12 (0.19) ***	[9.75, 10.49]	8.99 (0.32) ***	[8.36, 9.61]	10.14 (0.17) ***	[9.81, 10.47]	9.0 (0.27) ***	[8.47, 9.53]	10.19 (0.25) ***	[9.70, 10.68]	9.34 (0.40) ***	[8.55, 10.14]
Age (+1SD)			−0.83 (0.19) ***	[−1.21, −0.44]			−0.87 (0.16) ***	[−1.19, −0.56]			−0.51 (0.29) †	[−1.08, 0.07]
Female			1.07 (0.35) **	[0.38, 1.75]			1.13 (0.30) ***	[0.54, 1.73]			0.92 (0.51) †	[−0.08, 1.92]
Hispanic			−0.69 (0.54)	[−1.75, 0.37]			−0.58 (0.47)	[−1.51, 0.35]			−1.21 (1.05)	[−3.28, 0.86]
Nonwhite			−0.97 (0.41) *	[−1.77, −0.17]			−0.58 (0.38)	[−1.32, 0.16]			−0.27 (0.55)	[−1.36, 0.82]
Some College			1.62 (0.37) ***	[0.89, 2.36]			1.58 (0.32) ***	[0.94, 2.21]			1.29 (0.49) **	[0.32, 2.25]
Health			0.61 (0.16) ***	[0.30, 0.93]			0.50 (0.15) ***	[0.21, 0.79]			0.84 (0.22) ***	[0.40, 1.28]
Age of Onset (+1SD)	−0.25 (0.15) †	[−0.55, 0.05]	−0.06 (0.15)	[−0.35, 0.23]	−0.28 (0.15) †	[−0.58, 0.02]	−0.05 (0.15)	[−0.34, 0.23]	−0.02 (0.14)	[−0.30, 0.27]	0.004 (0.14)	[−0.27, 0.27]
Model Statistics												
F (DF)	2.75 (1, 314) †		11.40 (7, 305) ***		3.43 (1, 397) †		14.91 (7, 391) ***		0.01 (1, 169)		5.42 (7, 161) ***	
R Square	0.01		0.21		0.01		0.21		0.00		0.19	
Omega−Square	0.01	[0.00, 0.04]	0.19	[0.12, 0.27]	0.01	[0.00, 0.04]	0.20	[0.13, 0.27]	0.01	[0.00, 0.01]	0.15	[0.06, 0.26]

Note. † $p < 0.10$, * $p < 0.05$, ** $p < 0.01$, *** $p < 0.001$.

Table S3. Hierarchical regression analyses for presence of memory gap from TBI predicting self−rated memory in people without dementia.

**Variable**	**TBI from a Vehicle Accident**				**TBI from a Fall**				**TBI from a Sport**			
	**Model 1: Unadjusted**		**Model 2: Sociodemographic and Health Adjustment**		**Model 1: Unadjusted**		**Model 2: Sociodemographic and Health Adjustment**		**Model 1: Unadjusted**		**Model 2: Sociodemographic and Health Adjustment**	
	**B (SE)**	**95% CI**	**B (SE)**	**95% CI**	**B (SE)**	**95% CI**	**B (SE)**	**95% CI**	**B (SE)**	**95% CI**	**B (SE)**	**95% CI**
Intercept	2.12 (0.07) ***	[1.99, 2.25]	1.98 (0.10) ***	[1.78, 2.18]	2.10 (0.06) ***	[1.97, 2.22]	2.0 (0.09) ***	[1.82, 2.18]	2.21 (0.09) ***	[2.03, 2.39]	2.16 (0.13) ***	[1.89, 2.43]
Age (+1SD)			0.03 (0.05)	[−0.07, 0.13]			−0.02 (0.04)	[−0.11, 0.06]			−0.06 (0.08)	[−0.21, 0.10]
Female			−0.03 (0.10)	[−0.23, 0.16]			−0.09 (0.09)	[−0.26, 0.08]			−0.06 (0.14)	[−0.34, 0.22]
Hispanic			−0.08 (0.14)	[−0.36, 0.21]			0.24 (0.14) †	[−0.03, 0.51]			0.61 (0.29) *	[0.05, 1.17]
Nonwhite			0.11 (0.11)	[−0.11, 0.33]			0.04 (0.11)	[−0.18, 0.25]			−0.01 (0.15)	[−0.31, 0.30]
Some College			0.31 (0.10) **	[0.11, 0.51]			0.17 (0.09) †	[−0.01, 0.36]			0.07 (0.14)	[−0.19, 0.34]
Health			0.23 (0.04) ***	[0.15, 0.32]			0.25 (0.04) ***	[0.17, 0.33]			0.26 (0.06) ***	[0.14, 0.38]
Memory Gap	−0.20 (0.10) *	[−0.40, −0.01]	−0. 13 (0.10)	[−0.32, 0.07]	−0.19 (0.09) *	[−0.37, −0.02]	−0.10 (0.09)	[−0.28, 0.07]	−0.41 (0.13) **	[−0.67, −0.15]	−0.43 (0.13) **	[−0.68, −0.17]
Model Statistics												
F (DF)	4.17 (1, 312) *		6.80 (7, 303) ***		4.66 (1, 389) *		7.57 (7, 383) ***		9.57 (1, 168)		5.56 (7, 160) ***	
R Square	0.01		0.14		0.01		0.12		0.05		0.20	
Omega−Square	0.01	[0.00, 0.05]	0.12	[0.06, 0.19]	0.01	[0.00, 0.04]	0.11	[0.05, 0.17]	0.05	[0.01, 0.13]	0.16	[0.07, 0.27]

Note. † p < 0.10, * p < 0.05, ** p < 0.01, *** p < 0.001.

Table S4. Hierarchical regression analyses for presence of memory gap from TBI predicting recall memory performance in people without dementia.

Variable	TBI from a Vehicle Accident				TBI from a Fall				TBI from a Sport			
	**Model 1: Unadjusted**		**Model 2: Sociodemographic and Health Adjustment**		**Model 1: Unadjusted**		**Model 2: Sociodemographic and Health Adjustment**		**Model 1: Unadjusted**		**Model 2: Sociodemographic and Health Adjustment**	
	**B (SE)**	**95% CI**	**B (SE)**	**95% CI**	**B (SE)**	**95% CI**	**B (SE)**	**95% CI**	**B (SE)**	**95% CI**	**B (SE)**	**95% CI**
Intercept	10.43 (0.24) ***	[9.95, 10.91]	9.35 (0.37) ***	[8.63, 10.07]	10.04 (0.24) ***	[9.57, 10.50]	8.71 (0.32) ***	[8.07, 9.34]	10.62 (0.34) ***	[9.95, 11.28]	9.62 (0.48) ***	[8.67, 10.56]
Age (+1SD)			−0.91 (0.18) ***	[−1.28, −0.55]			−0.87 (0.15) ***	[−1.17, −0.56]			−0.49 (0.29) †	[−1.05, 0.08]
Female			0.92 (0.35) **	[0.23, 1.60]			1.14 (0.30) ***	[0.54, 1.74]			0.70 (0.51)	[−0.30, 1.70]
Hispanic			−0.59 (0.52)	[−1.60, 0.43]			−0.62 (0.48)	[−1.56, 0.31]			−1.01 (1.02)	[−3.02, 1.004]
Nonwhite			−1.14 (0.40) **	[−1.93, −0.34]			−0.57 (0.38)	[−1.31, 0.17]			−0.11 (0.55)	[−1.20, 0.97]
Some College			1.50 (0.37) ***	[0.78, 2.22]			1.71 (0.33) ***	[1.07, 2.35]			1.24 (0.49) *	[0.28, 2.20]
Health			0.55 (0.16) ***	[0.24, 0.86]			0.50 (0.15) ***	[0.21, 0.79]			0.92 (0.22) ***	[0.49, 1.36]
Memory Gap	−0.61 (0.37)	[−1.35, 0.13]	−0.44 (0.35)	[−1.14, 0.25]	0.24 (0.34)	[−0.42, 0.90]	0.54 (0.31) †	[−0.07, 1.14]	−0.94 (0.49) †	[−1.90, 0.03]	−0.57 (0.47)	[−1.49, 0.36]
Model Statistics												
F (DF)	2.64 (1, 310)		11.37 (7, 301) ***		0.52 (1, 391)		15.12 (7, 385) ***		3.70 (1, 168) *		6.18 (7, 160) ***	
R Square	0.01		0.21		0.001		0.22		0.02		0.21	
Omega−Square	0.01	[0.00, 0.04]	0.19	[0.12, 0.27]	0.00	[0.00, 0.02]	0.20	[0.14, 0.27]	0.02	[0.00, 0.08]	0.18	[0.08, 0.29]

Note. † $p < 0.10$, * $p < 0.05$, ** $p < 0.01$, *** $p < 0.001$.

Table S5. Hierarchical regression analyses for TBI age of onset predicting self−rated memory in people without Alzheimer's disease.

Variable	TBI from a Vehicle Accident				TBI from a Fall				TBI from a Sport			
	Model 1: Unadjusted		Model 2: Sociodemographic and Health Adjustment		Model 1: Unadjusted		Model 2: Sociodemographic and Health Adjustment		Model 1: Unadjusted		Model 2: Sociodemographic and Health Adjustment	
	B (SE)	95% CI	B (SE)	95% CI	B (SE)	95% CI	B (SE)	95% CI	B (SE)	95% CI	B (SE)	95% CI
Intercept	2.02 (0.05) ***	[1.92, 2.12]	1.87 (0.09) ***	[1.70, 2.04]	1.96 (0.04) ***	[1.87, 2.06]	1.88 (0.08) ***	[1.73, 2.03]	1.98 (0.07) ***	[1.85, 2.12]	1.92 (0.11) ***	[1.70, 2.14]
Age (+1SD)			0.06 (0.05)	[−0.05, 0.16]			−0.002 (0.04)	[−0.09, 0.09]			−0.01 (0.08)	[−0.16, 0.15]
Female			0.02 (0.10)	[−0.17, 0.21]			−0.03 (0.09)	[−0.20, 0.14]			−0.07 (0.14)	[−0.35, 0.21]
Hispanic			−0.03 (0.15)	[−0.32, 0.27]			0.27 (0.14) *	[0.01, 0.54]			0.67 (0.30) *	[0.09, 1.26]
Nonwhite			0.12 (0.11)	[−0.10, 0.34]			0.05 (0.11)	[−0.17, 0.26]			0.02 (0.16)	[−0.30, 0.32]
Some College			0.34 (0.10) **	[0.14, 0.54]			0.17 (0.10) †	[−0.01, 0.35]			0.14 (0.14)	[−0.13, 0.41]
Health			0.26 (0.04) ***	[0.17, 0.35]			0.28 (0.04) ***	[0.19, 0.36]			0.32 (0.06) ***	[0.20, 0.44]
Age of Onset (+1SD)	0.01 (0.04)	[−0.07, 0.09]	−0.01 (0.04)	[−0.09, 0.07]	−0.11 (0.04) **	[−0.19, −0.03]	−0.07 (0.04)	[−0.15, 0.1]	−0.05 (0.04)	[−0.12, 0.02]	−0.07 (0.04) †	[−0.14, 0.00]
Model Statistics												
F (DF)	0.02 (1, 324)		7.80 (7, 315) ***		7.06 (1, 408) **		9.33 (7, 402) ***		1.87 (1, 174)		5.75 (7, 166) ***	
R Square	0.0001		0.15		0.02		0.14		0.01		0.20	
Omega−Square	0.00	[0.00, 0.01]	0.13	[0.07, 0.20]	0.01	[0.00, 0.05]	0.12	[0.07, 0.19]	0.00	[0.00, 0.06]	0.16	[0.07, 0.27]

Note. † $p < 0.10$, * $p < 0.05$, ** $p < 0.01$, *** $p < 0.001$.

Table S6. Hierarchical regression analyses for TBI age of onset predicting recall memory performance in people without Alzheimer's disease.

Variable	TBI from a Vehicle Accident				TBI from a Fall				TBI from a Sport			
	**Model 1: Unadjusted**		**Model 2: Sociodemographic and Health Adjustment**		**Model 1: Unadjusted**		**Model 2: Sociodemographic and Health Adjustment**		**Model 1: Unadjusted**		**Model 2: Sociodemographic and Health Adjustment**	
	**B(SE)**	**95% CI**	**B (SE)**	**95% CI**	**B (SE)**	**95% CI**	**B (SE)**	**95% CI**	**B (SE)**	**95% CI**	**B (SE)**	**95% CI**
Intercept	10.05 (0.19) ***	[9.68, 10.43]	8.90 (0.32) ***	[8.27, 9.54]	10.02 (0.17) ***	[9.66, 10.35]	8.81 (0.27) ***	[8.28, 9.34]	10.03 (0.25) ***	[9.54, 10.53]	9.26 (0.40) ***	[8.47, 10.04]
Age (+1SD)			−0.77 (0.20) ***	[−1.16, −0.40]			−0.86 (0.16) ***	[−1.17, −0.54]			−0.51 (0.28) *	[−1.07, 0.04]
Female			1.08 (0.35) **	[0.38, 1.78]			1.17 (0.31) ***	[0.57, 1.77]			0.61 (0.51)	[−0.39, 1.61]
Hispanic			−0.60 (0.55)	[−1.69, 0.49]			−0.43 (0.48)	[−1.38, 0.52]			−0.79 (1.06)	[−2.89, 1.31]
Nonwhite			−1.00 (0.41) *	[−1.81, −0.19]			−0.56 (0.38)	[−1.31, 0.19]			−0.27 (0.56)	[−1.37, 0.84]
Some College			1.75 (0.38) ***	[1.00, 2.50]			1.72 (0.33) ***	[1.08, 2.36]			1.38 (0.49) **	[0.40, 2.36]
Health			0.65 (0.16) ***	[0.34, 0.97]			0.57 (0.15) ***	[0.28, 0.86]			0.97 (0.22) ***	[0.53, 1.41]
Age of Onset (+1SD)	−0.21 (0.16)	[−0.52, 0.09]	−0.04 (0.15)	[−0.33, 0.26]	−0.29 (0.15) †	[−0.59, 0.01]	−0.07 (0.15)	[−0.36, 0.22]	−0.15 (0.14)	[−0.42, 0.12]	−0.13 (0.13)	[−0.38, 0.12]
Model Statistics												
F (DF)	1.90 (1, 323)		11.57 (7, 314) ***		3.56 (1, 410) †		16.03 (7, 404) ***		1.15 (1, 174)		6.35 (7, 166) ***	
R Square	0.01		0.20		0.01		0.22		0.01		0.21	
Omega−Square	0.00	[0.00, 0.03]	0.19	[0.12, 0.26]	0.01	[0.00, 0.03]	0.20	[0.14, 0.27]	0.00	[0.00, 0.05]	0.18	[0.08, 0.29]

Note. † p < 0.10, * p < 0.05, ** p < 0.01, *** p < 0.001.

Table S7. Hierarchical regression analyses for presence of memory gap from TBI predicting self−rated memory in people without Alzheimer's disease.

Variable	TBI from a Vehicle Accident				TBI from a Fall				TBI from a Sport			
	**Model 1: Unadjusted**		**Model 2: Sociodemographic and Health Adjustment**		**Model 1: Unadjusted**		**Model 2: Sociodemographic and Health Adjustment**		**Model 1: Unadjusted**		**Model 2: Sociodemographic and Health Adjustment**	
	**B (SE)**	**95% CI**	**B (SE)**	**95% CI**	**B (SE)**	**95% CI**	**B (SE)**	**95% CI**	**B (SE)**	**95% CI**	**B (SE)**	**95% CI**
Intercept	2.12 (0.07) ***	[1.99, 2.25]	2.00 (0.10) ***	[1.80, 2.20]	2.07 (0.06) ***	[1.95, 2.20]	1.96 (0.09) ***	[1.77, 2.14]	2.20 (0.09) ***	[2.02, 2.38]	2.18 (0.13) ***	[1.92, 2.44]
Age (+1SD)			0.04 (0.05)	[−0.06, 0.14]			−0.03 (0.04)	[−0.11, 0.06]			−0.02 (0.08)	[−0.17, 0.13]
Female			−0.05 (0.10)	[−0.24, 0.14]			−0.07 (0.09)	[−0.24, 0.10]			−0.10 (0.14)	[−0.38, 0.17]
Hispanic			−0.07 (0.14)	[−0.35, 0.22]			0.27 (0.14) *	[0.000, 0.54]			0.64 (0.29) *	[0.07, 1.20]
Nonwhite			0.09 (0.11)	[−0.13, 0.31]			0.06 (0.11)	[−0.15, 0.27]			0.005 (0.15)	[−0.30, 0.31]
Some College			0.31 (0.10) **	[0.11, 0.51]			0.18 (0.09) †	[−0.003, 0.36]			0.07 (0.14)	[−0.20, 0.34]
Health			0.24 (0.04) ***	[0.16, 0.33]			0.27 (0.04) ***	[0.19, 0.36]			0.28 (0.06) ***	[0.16, 0.40]
Memory Gap	−0.22 (0.10) *	[−0.42, −0.03]	−0.14 (0.10)	[−0.33, 0.05]	−0.23 (0.09) *	[−0.41, −0.05]	−0.13 (0.09)	[−0.30, 0.05]	−0.40 (0.13) **	[−0.66, −0.14]	−0.42 (0.13) **	[−0.68, −0.17]
Model Statistics												
F (DF)	4.93 (1, 319) *		7.53 (7, 310) ***		6.54 (1, 402) *		9.03 (7, 396) ***		9.17 (1, 172) **		6.07 (7, 164) ***	
R Square	0.02		0.15		0.02		0.14		0.05		0.21	
Omega−Square	0.01	[0.00, 0.05]	0.13	[0.06, 0.20]	0.01	[0.00, 0.05]	0.12	[0.07, 0.19]	0.04	[0.01, 0.13]	0.17	[0.08, 0.28]

Note. † $p < 0.10$, * $p < 0.05$, ** $p < 0.01$, *** $p < 0.001$.

Table S8. Hierarchical regression analyses for presence of memory gap from TBI predicting recall memory performance in people without Alzheimer's disease.

**Variable**	**TBI from a Vehicle Accident**				**TBI from a Fall**				**TBI from a Sport**			
	**Model 1: Unadjusted**		**Model 2: Sociodemographic and Health Adjustment**		**Model 1: Unadjusted**		**Model 2: Sociodemographic and Health Adjustment**		**Model 1: Unadjusted**		**Model 2: Sociodemographic and Health Adjustment**	
	**B (SE)**	**95% CI**	**B (SE)**	**95% CI**	**B (SE)**	**95% CI**	**B (SE)**	**95% CI**	**B (SE)**	**95% CI**	**B (SE)**	**95% CI**
Intercept	10.41 (0.25) ***	[9.92, 10.89]	9.37 (0.37) ***	[8.64, 10.10]	9.96 (0.24) ***	[9.48, 10.43]	8.54 (0.33) ***	[7.89, 9.18]	10.53 (0.34) ***	[9.85, 11.21]	9.56 (0.48) ***	[8.61, 10.52]
Age (+1SD)			−0.88 (0.18) ***	[−1.24, −0.52]			−0.85 (0.16) ***	[−1.16, −0.55]			−0.51 (0.28) †	[−1.06, 0.04]
Female			0.85 (0.35) *	[0.16, 1.55]			1.17 (0.31) ***	[0.56, 1.77]			0.48 (0.51)	[−0.53, 1.49]
Hispanic			−0.51 (0.52)	[−1.54, 0.51]			−0.47 (0.49)	[−1.43, 0.49]			−0.83 (1.04)	[−2.88, 1.21]
Nonwhite			−1.25 (0.41) **	[−2.05, −0.45]			−0.54 (0.38)	[−1.29, 0.21]			−0.08 (0.55)	[−1.17, 1.01]
Some College			1.59 (0.37) ***	[0.86, 2.32]			1.85 (0.33) ***	[1.20, 2.50]			1.33 (0.49) **	[0.36, 2.30]
Health			0.60 (0.16) ***	[0.29, 0.91]			0.57 (0.15) ***	[0.28, 0.87]			1.01 (0.22) ***	[0.57, 1.45]
Memory Gap	−0.63 (0.38) †	[−1.37, 0.11]	−0.46 (0.35)	[−1.15, 0.24]	0.14 (0.34)	[−0.53, 0.81]	0.48 (0.31)	[−0.13, 1.09]	−1.00 (0.50) *	[−1.98, −0.02]	−0.63 (0.47)	[−1.56, 0.29]
Model Statistics												
F (DF)	2.82 (1, 317) †		11.75 (7, 308) ***		0.18 (1, 404)		16.06 (7, 398) ***		4.05 (1, 172) *		6.89 (7, 164) ***	
R Square	0.01		0.21		0.00		0.22		0.02		0.23	
Omega−Square	0.01	[0.00, 0.04]	0.19	[0.12, 0.27]	−0.00	[0.00, 0.01]	0.21	[0.14, 0.28]	0.02	[0.00, 0.08]	0.19	[0.10, 0.30]

Note. † $p < 0.10$, * $p < 0.05$, ** $p < 0.01$, *** $p < 0.001$.
